# Metabolic healthy obesity is associated with higher incidence of mild decrease estimate glomerular rate in rural northeast Chinese

**DOI:** 10.1186/s12882-020-02164-2

**Published:** 2020-11-24

**Authors:** Shasha Yu, Xiaofan Guo, Guang Xiao Li, Hongmei Yang, Liqiang Zheng, Yingxian Sun

**Affiliations:** 1 Department of Cardiology, First Hospital of China Medical University, Shenyang, 110001 China; 2 Department of Clinical Epidemiology, Institute of Cardiovascular Diseases, First Hospital of China Medical University, Shenyang, 110001 China; 3 Department of Clinical Epidemiology, Shengjing Hospital of China Medical University, Shenyang, 110004 China

## Abstract

**Background:**

Metabolic healthy obesity (MHO), a phenotype of obesity, seems to be associated with a lower risk of cardiovascular disease. However, MHO has a close relationship with a higher incidence of metabolic syndrome and diabetes. This study aimed to investigate the prevalence of MHO at baseline, the changes in the obese metabolic phenotype at follow-up and the relationship of this phenotype with the incidence of mildly reduced estimated glomerular filtration rate (eGFR) in rural Northeast Chinese.

**Methods:**

The Chronic Kidney Disease Epidemiology (CKD-EPI) equation was used to calculate eGFR. A total of 4903 participants aged ≥35 years with eGFR > 90 ml/min/1.73 m^2^ at baseline were enrolled and successfully followed. All participants completed the questionnaires, anthropometric measurements, and blood tests during baseline and follow-up. Mild renal dysfunction was defined as mildly reduced eGFR between 60 and 90 ml/min/1.73 m^2^.

**Results:**

The prevalence of MHO was 20.0% at baseline (19.0% for women and 21.1% for men), which was secondary to metabolic abnormal obesity (MAO) (24.4, 27.2% for women and 21.5% for men). A total of 38.4% of women and 38.9% of men experienced phenotypic changes during follow-up. The cumulative incidence of mildly reduced eGFR in the MHO group was 20.1% (17.7% for women and 22.3% for men), which was also secondary to the incidence in the MAO group (20.8, 18.6% for women and 23.5% for men). After adjusting for age, current smoking, current drinking, chronic diseases, LDL-C, ALT, and AST, MHO was associated with a higher incidence of mildly reduced eGFR among women [OR (95% CI) =1.6 (1.2, 2.3)] and men [OR (95% CI) =1.6(1.2, 2.1)], whereas MAO was related to a higher incidence of mildly reduced eGFR among men only [OR (95% CI) =1.7 (1.3, 2.3)].

**Conclusion:**

MHO was associated with a higher incidence of mildly reduced eGFR in both sexes; however, there was a specific relationship between MAO and mildly reduced eGFR in men only. Therefore, it is necessary to monitor kidney function among participants with both MHO and MAO.

## Background

Mildly impaired renal function is used to define subjects with either mildly reduced estimated glomerular filtration rate (eGFR) or microalbuminuria. The National Health and Nutrition Examination Surveys (NHANES), conducted in the United States, enrolled 20- to 75-year-old subjects and reported that approximately 36% had an eGFR of 60 to 89 ml/min/1.73 m^2^, whereas in the Atherosclerosis Risk in the Communities (ARIC) study, 50% of participants aged 45 to 64 years had a mild reduction in eGFR [1, 2]. Many studies have claimed that mildly reduced eGFR is associated with an increased risk of cardiovascular diseases [1–4]. Furthermore, evidence indicated that when treating cardiovascular risk factors, patients with a mild reduction in eGFR experienced a reduction in cardiovascular events and progression of renal disease [5]. Therefore, it is important to determine the possible risk factors for mildly reduced eGFR to better control its complications.

Accumulative evidence indicates that obesity is becoming increasingly prevalent among rural residents worldwide [6, 7]. A study enrolled rural residents from Nepal and reported that 27% of males and 72% of females were obese [8]. Data from rural India showed that in 2008, 10.1% of men and 14.6% of women were overweight (including obesity), whereas 17.3% of men and 24.7% of women were overweight in 2017 [9]. There is a higher rate of obesity in rural areas (37.7% vs. 32.5% for men; 33.4% vs. 28.2% for women) than in urban areas in the USA [10]. Similarly, the prevalence of obesity in 15.8 million men in rural China was 33.3% [11], whereas the prevalence of obesity among 1.37 million rural Chinese women was 38.4% [12]. Obesity is associated with increasing mortality and a high prevalence of metabolic disorders. Obesity has been confirmed as an important cause of kidney disease due to its close association with diabetes and hypertension [13]. However, 10–30% of obese subjects lack abnormal blood pressure or lipid profiles, indicating that a certain proportion of obese subjects are in a relatively healthy metabolic status [14, 15]. There are studies that have reported that metabolic healthy obesity (MHO) was associated with lower mortality and participants with MHO had a lower risk of developing metabolic diseases than participants with metabolic abnormal obesity (MAO) [16, 17]. However, there is a lack of data to evaluate the possible effect of MHO on the newly diagnosed mildly reduced eGFR. Hence, in the present study, we first estimated the prevalence of the obese phenotype at baseline, the changes in the obese metabolic phenotype over time, and the cumulative incidence of mildly reduced eGFR at follow-up. Second, we aimed to determine the possible relationship between MHO and mildly reduced eGFR among rural Northeast Chinese individuals.

## Methods

### Study population

**Fig. 1:**
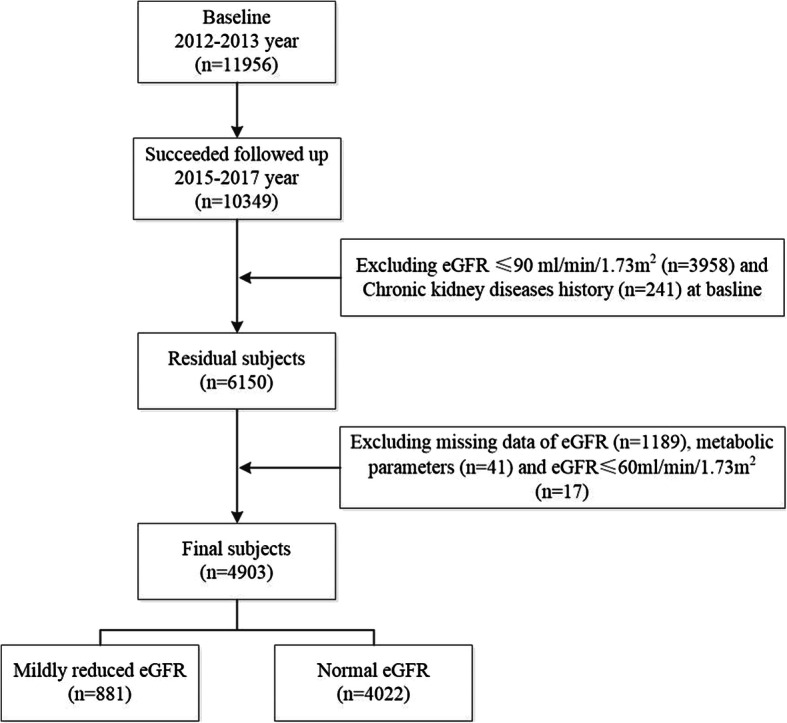
Flow chart of participants included in this study after inclusion and exclusion

The Northeast China Rural Cardiovascular Health Study (NCRCHS) is a community-based prospective cohort study carried out in rural areas of Northeast China. The design and inclusion criteria of the study have been described previously [18, 19]. In brief, a total of 11,956 participants aged ≥35 years were recruited from Dawa, Zhangwu and Liaoyang counties in Liaoning Province between 2012 and 2013 using a multistage, randomly stratified cluster-sampling scheme. In total, 26 rural villages were included. All eligible permanent residents (aged ≥35 years) from each village were invited to participate in the study, comprising a potential pool of 14,016 people. Of these, 11,956 participants agreed and completed the present study, yielding a response rate of 85.3%. Participants who were pregnant, had a malignant tumor, or had mental disorders were excluded. The study was approved by the Ethics Committee of China Medical University (Shenyang, China AF-SDP-07-1, 0–01). Detailed information was collected at baseline for each participant. In 2015 and 2017, participants were invited to attend a follow-up study. Of the 11,956 participants, 1256 participants were not included due to missing contact information, and 10,349 participants (86.6%) completed at least one follow-up visit. The median follow-up was 4.66 years. Written informed consent was obtained from all participants. The detailed recruitment and selection process of participants is shown in Fig. 1.

### Study variables

At baseline, detailed information on demographic characteristics and medical history was obtained by interview using a standardized questionnaire. Smoking and drinking status were defined as current use (yes or no). Dietary pattern included were assessed by residents recall the foods that they eat in the previous year. The average consumption of several food items per week was recorded through the questionnaire. The reported consumption was quantified approximately in terms of grams per week (Vegetable consumption: rarely = 3, < 1000 g = 2, 1000–2000 g = 1, ≥2000 g = 0; meat consumption including red meat, fish, and poultry: rarely = 0, < 250 g = 1, 250–500 g = 2, ≥500 g = 3). The diet score was calculated for each participant as the vegetable consumption score plus the meat consumption score (range: 0–6) as previous study [20]. History of stroke, CHD and heart failure at baseline was self-reported and confirmed by medical records. Weight and height were measured with participants in lightweight clothing and without shoes. Waist circumference was measured at the umbilicus using a non-elastic tape. Body mass index (BMI) was computed as weight in kilograms divided by the square of height in metres. Blood pressure was assessed three times with participants seated after at least 5 min of rest using a standardized automatic electronic sphygmomanometer (HEM-907; Omron, Tokyo, Japan). Hypertension was defined as systolic blood pressure (SBP) ≥140 mmHg and/or diastolic blood pressure (DBP) ≥ 90 mmHg and/or use of antihypertensive medications [21]. Fasting blood samples were collected in the morning from participants who had fasted for at least 12 h. Fasting plasma glucose (FPG), total cholesterol (TC), low-density lipoprotein cholesterol (LDL-C), high-density lipoprotein cholesterol (HDL-C), triglycerides (TG), serum creatinine and other routine blood biochemical indexes were analysed enzymatically.

### Definition

The estimated glomerular filtration rate (eGFR) was calculated using the Chronic Kidney Disease Epidemiology Collaboration (CKD-EPI) equation [22]. Mildly reduced eGFR was defined as eGFR between 60 and 90 ml/min/1.73 m^2^. According to the World Health Organization Asia Pacific guidelines, BMI ≥ 25 kg/m^2^ was defined as obesity [23]. Metabolic syndrome (MetS) was diagnosed following the unifying criteria from the meeting between several major organizations in 2009 [24]; the presence of any 3 of 5 risk factors constitutes a diagnosis of metabolic syndrome: 1. Elevated waist circumference (population- and country-specific definitions): ≥90 cm for men; ≥80 cm for women (Asians; Japanese; South and Central Americans); 2. Elevated triglycerides (drug treatment for elevated triglycerides is an alternate indicator): ≥150 mg/dL (1.7 mmol/L); 3. Reduced HDL-C (drug treatment for reduced HDL-C is an alternate indicator): < 40 mg/dL (1.0 mmol/L) in men; < 50 mg/dL (1.3 mmol/L) in women; 4. Elevated blood pressure (antihypertensive drug treatment in a patient with a history of hypertension is an alternate indicator): systolic ≥130 and/or diastolic ≥85 mmHg; and 5. Elevated fasting glucose (drug treatment of elevated glucose is an alternate indicator): ≥100 mg/dL. MHO was considered obesity with an absence of MetS [25]. Metabolically healthy non-obesity (MHNO) was defined as the absence of MetS and obesity. Metabolically abnormal non-obesity (MANO) and metabolically abnormal obesity (MAO) were defined as MetS coexisting with or without obesity, respectively.

### Statistical analysis

Descriptive statistics were calculated for all the variables, including continuous variables (reported as the mean values and standard deviations) and categorical variables (reported as numbers and percentages). Differences among categories were evaluated using t-tests, ANOVA, ANCOVA, non-parametric tests or the χ2-test as appropriate. We used logistic regression analyses to estimate odds ratios (ORs) and 95% confidence intervals (CIs) for the evaluation of the relationship between the obese phenotype and mildly reduced eGFR after adjusting for possible confounders. All statistical analyses were performed using SPSS version 17.0 software (Chicago, IL), and *P* values less than 0.05 were considered statistically significant.

## Results

### Baseline character of newly diagnosed mildly reduced eGFR

Table 1 showed that residents with newly diagnosed mildly reduced eGFR were older and had higher values of SBP, DBP, BMI, WC and HDL-C but lower eGFR than participants with normal eGFR at baseline. In addition, participants with mildly reduced eGFR tended to have a higher rate of current smoking but not drinking at baseline.

**Table 1:** Baseline characters of mildly reduced eGFR subjects

Characteristics	Total			Female			Male		
	eGFR 60–90 (***n*** = 881)	eGFR > 90 (***n*** = 4022)	***P***-value	eGFR 60–90 (***n*** = 400)	eGFR > 90 (***n*** = 235)	***P***-value	eGFR 60–90 (***n*** = 481)	eGFR > 90 (***n*** = 1987)	***P***-value
**Age (years)**	55.28 ± 9.08	49.42 ± 8.00	< 0.001	55.46 ± 8.86	48.57 ± 7.69	< 0.001	55.13 ± 9.26	50.29 ± 8.23	< 0.001
**SBP (mmHg) (C.V)**	150.38 ± 26.12(17.4)	138.76 ± 21.37(15.4)	< 0.001	150.91 ± 26.99 (17.9)	137.16 ± 22.07 (16.1)	< 0.001	149.93 ± 25.39 (16.9)	140.40 ± 20.50 (14.6)	< 0.001
**DBP (mmHg) (C.V)**	83.27 ± 12.33 (14.8)	81.66 ± 11.23 (13.8)	< 0.001	81.21 ± 12.03 (14.8)	80.04 ± 10.70 (13.4)	0.050	84.98 ± 12.32 (14.5)	83.33 ± 11.52 (13.8)	0.005
**BMI (kg/m**^**2**^**) (C.V)**	25.30 ± 3.49 (13.8)	24.75 ± 3.74 (15.1)	< 0.001	25.32 ± 3.73 (14.7)	24.93 ± 3.86 (15.5)	0.065	25.28 ± 3.28 (13.0)	24.57 ± 3.60 (14.6)	< 0.001
**WC (cm) (C.V)**	83.61 ± 9.59 (11.5)	81.65 ± 9.65 (11.8)	< 0.001	81.92 ± 9.77 (11.9)	80.38 ± 9.57 (11.9)	0.003	85.03 ± 9.21 (10.8)	82.95 ± 9.57 (11.5)	< 0.001
**HbA**_**1c**_ **(%) (C.V)**	5.31 ± 0.69 (12.9)	5.34 ± 0.99 (18.7)	0.833	5.53 ± 0.68 (12.3)	5.30 ± 0.82 (15.4)	0.262	5.23 ± 0.68 (13.0)	5.37 ± 1.12 (20.8)	0.413
**TC (mmol/L) (C.V)**	5.17 ± 1.44 (20.2)	5.11 ± 1.02 (20.0)	0.088	5.27 ± 1.08 (20.6)	5.06 ± 1.03 (20.4)	< 0.001	5.09 ± 1.01 (19.8)	5.16 ± 1.00 (19.4)	0.213
**TG (mmol/L) (C.V)**	1.56 ± 1.74 (111.1)	1.53 ± 1.47 (95.8)	0.566	1.43 ± 0.87 (61.2)	1.45 ± 1.17 (80.0)	0.782	1.68 ± 2.21 (131.7)	1.62 ± 1.73 (106.6)	0.541
**LDL-C (mmol/L) (C.V)**	3.06 ± 0.89 (28.9)	2.86 ± 0.79 (27.7)	0.080	3.21 ± 0.93 (29.1)	2.86 ± 0.81 (28.4)	< 0.001	2.95 ± 0.82 (28.0)	2.86 ± 0.77 (26.9)	0.041
**HDL-C (mmol/L) (C.V)**	1.51 ± 0.42 (28.0)	1.45 ± 0.40 (27.4)	< 0.001	1.52 ± 0.37 (24.1)	1.44 ± 0.35 (24.3)	< 0.001	1.49 ± 0.46 (30.9)	1.46 ± 0.44 (30.1)	0.137
**FPG (mmol/L) (C.V)**	5.73 ± 1.44 (25.2)	5.84 ± 1.70 (29.1)	0.059	5.70 ± 1.59 (27.9)	5.75 ± 1.60 (27.9)	0.575	5.75 ± 1.31 (22.8)	5.95 ± 1.79 (30.2)	0.028
**eGFR (ml/min/1.73m**^**2**^**) (C.V)**	98.88 ± 9.69 (9.8)	103.04 ± 10.29 (10.0)	< 0.001	99.88 ± 12.68 (12.7)	103.62 ± 10.50 (10.1)	< 0.001	98.05 ± 6.07 (6.2)	102.44 ± 10.04 (9.8)	< 0.001
**Creatinine (μmol/L) (C.V)**	66.73 ± 11.40 (17.3)	65.31 ± 11.58 (17.8)	0.001	57.70 ± 8.13 (14.0)	57.77 ± 8.52 (14.6)	0.877	74.24 ± 7.68 (10.3)	73.03 ± 8.90 (12.1)	0.006
**BUN (mmol/L) (C.V)**	5.72 ± 1.87 (33.4)	5.26 ± 1.43 (28.1)	< 0.001	5.38 ± 2.22 (42.8)	4.86 ± 1.33 (28.5)	< 0.001	5.99 ± 1.47 (24.9)	5.67 ± 1.41 (25.8)	< 0.001
**Diet score**	2.43 ± 1.10	2.47 ± 1.08	0.346	2.24 ± 1.09	2.30 ± 1.06	0.362	2.59 ± 1.08	2.65 ± 1.06	0.280
**Current smoking (%)**	376 (42.7)	1517 (37.7)	0.004	90 (22.5)	285 (14.0)	< 0.001	286 (59.5)	1232 (62.0)	0.164
**Current drinking (%)**	253 (28.7)	1083 (26.9)	0.149	14 (3.5)	58 (2.9)	0.286	239 (49.7)	1025 (51.6)	0.243

Data are mean ± SD or number (%). *MHNO* metabolically health non-obese, *MHO* metabolically healthy obese, *MANO* metabolically abnormal obese, *MAO* metabolically abnormal obese, *SBP* systolic blood pressure, *DBP* diastolic blood pressure, *TC* total cholesterol, *TG* triglyceride, *LDL-C* low-density lipoprotein cholesterol, *HDL-C* high-density lipoprotein cholesterol, *FPG* fasting plasma glucose, *C.V* Coefficient of Variance, *BUN* blood urea nitrogen

BMI and FPG were higher among men with mildly reduced eGFR, while higher TC existed only among women. Furthermore, among women solely, the rate of current smoking was higher in those with mildly reduced eGFR.

### Prevalence of obese phenotype at baseline and cumulative incidence of mildly reduced eGFR among different obese phenotype

**Fig. 2:**
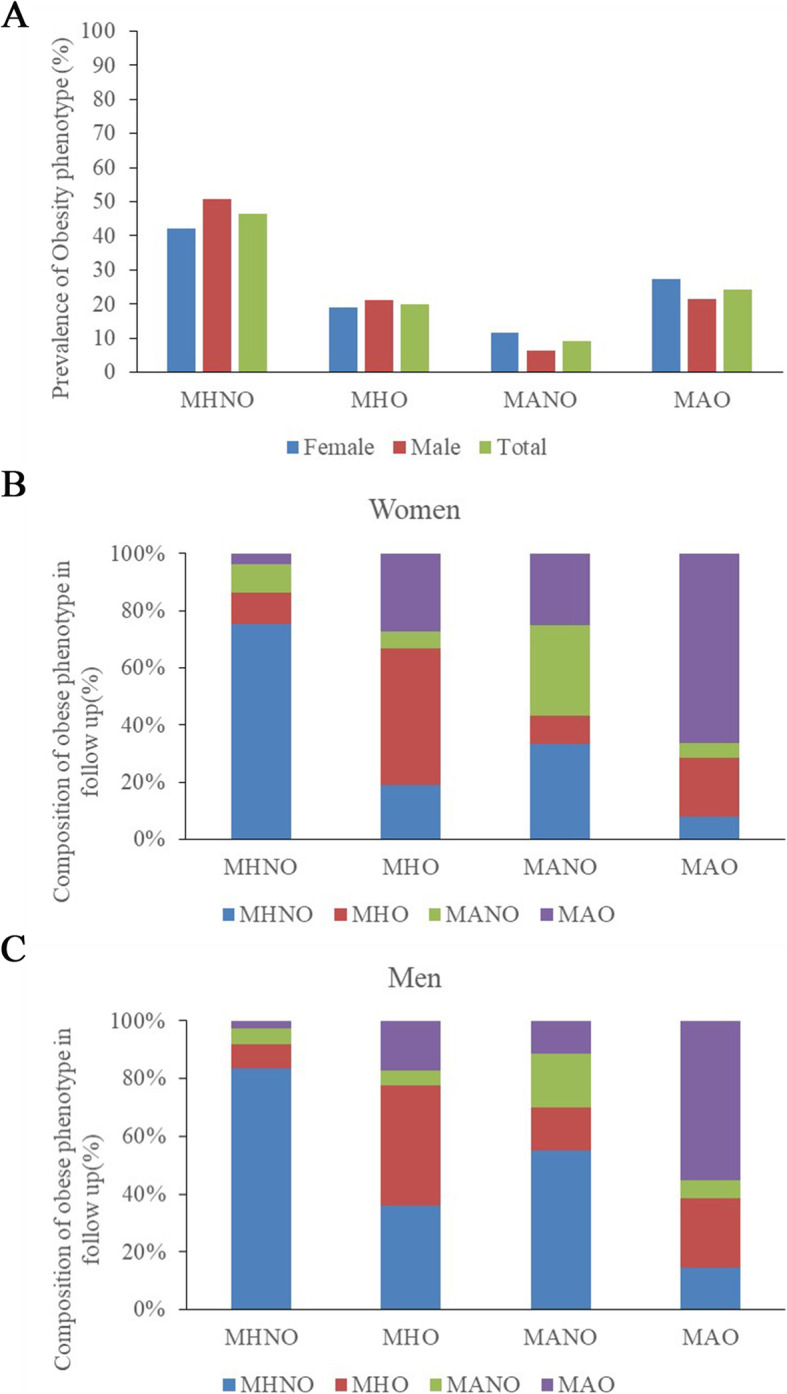
**a**. Prevalence of different obese phenotype at baseline. **b**, **c**. The changes of composition of obese phenotype in follow-up. MHNO metabolically health non-obese, MHO metabolically healthy obese, MANO metabolically abnormal obese, MAO metabolically abnormal obese

**Fig. 3:**
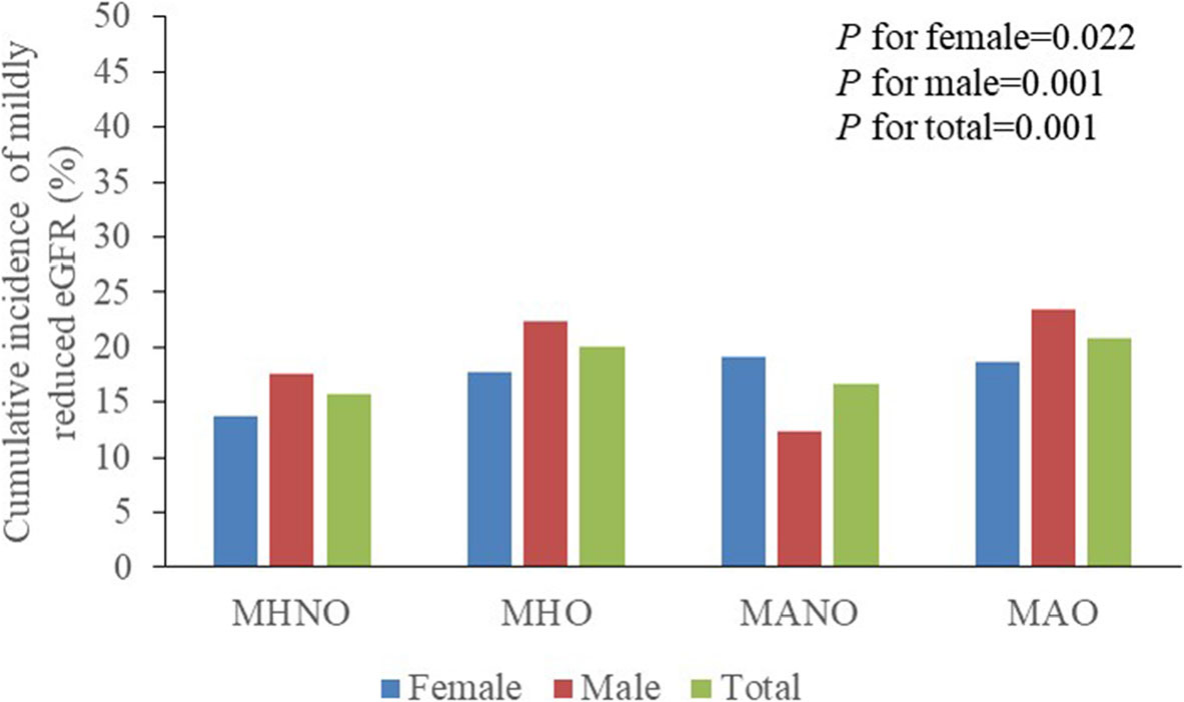
Cumulative incidence of mildly reduced eGFR among different obese phenotype at baseline. MHNO metabolically health non-obese, MHO metabolically healthy obese, MANO metabolically abnormal obese, MAO metabolically abnormal obese

Figure 2a shows that, in general, 46.5% of the residents were without MetS or obesity, 20.0% had MHO, 9.1% had MANO and 24.4% had MAO. There was a significant difference in the distribution of the obese phenotypes among women and men. There were fewer women than men who had neither MetS nor obesity. The rate of MAO was higher among women than men (27.3% vs. 21.5%). Figure 2b and c represent the changes in the obese metabolic phenotype over time. In all, 38.4% of women and 38.90% of men experienced phenotypic changes during follow-up. The MHO group had a higher proportion of transition to the MAO phenotype than the MHNO group in both women (28.26% vs. 4.99%) and men (32.3% vs. 8.09%). Figure 3 shows the cumulative incidence of mildly reduced eGFR among the different obese phenotypes; in general, the incidence rates among the different obese phenotypes were 15.8% in MHNO, 20.1% in MHO, 16.7% in MANO and 20.8% in MAO. There was an increasing trend in incidence among those with either MetS or obesity. The cumulative incidence showed a significant difference among women and men. In women, the highest incidence of mildly reduced eGFR was among the MANO group, while in women, it was among the men in the MAO group. In addition, among men but not women, the incidence of mildly reduced eGFR seemed relatively lower in the MANO group compared to the MHNO group; there was a sex discrepancy in the incidence among obese phenotypes.

### Changes of metabolic parameters of different obese phenotype from 2012 to 2013 to 2015–2017

Table 2 shows the changes in different metabolic parameters in the obese phenotype. SBP, LDL-C, HDL-C and eGFR significantly decreased at follow-up, whereas WC and TG increased at follow-up. BMI and DBP both increased and decreased among the different obese phenotypes. The changes in the metabolic parameters also differed by sex. The changes in DBP significantly varied among the different obese phenotypes in men but not in women, whereas changes in HDL-C showed variation among women but not among men. The changes in DBP were greater in the MHO group than in the MHNO group, whereas changes in BMI and WC were higher in the men with MHNO. Among women, changes in SBP, BMI, and HDL-C were greater in the MHO group than in the MHNO group. Table 3 shows the metabolic parameters at follow-up in the different metabolic phenotype groups after adjusting for baseline value. The data showed that subjects who had MHO had significantly higher values of DBP, BMI, WC, TC, and TG but lower HDL-C and eGFR compared those with MHNO. Similarly, MANO had relatively higher values of TG and FPG and lower values of HDL-C compared those with MHNO. Notably, the subjects with MAO had higher values of almost all metabolic parameters. In addition, we subdivided the participants by sex; and the values of the different metabolic parameters are presented in Table 3.

**Table 2:** Changes of metabolic parameters of metabolic health non-obese (MHNO), metabolically healthy obese (MHO), metabolic abnormal non-obese (MANO) and metabolic abnormal obese (MAO) subjects from 2012 to 2013 to 2015–2017

	Total					Men				Women					***P***-value
	MHNO	MHO	MANO	MAO	***P***-value	MHNO	MHO	MANO	MAO	***P***-value	MHNO	MHO	MANO	MAO	
**△SBP (mmHg)**	−4.51	−6.14*	−8.43*^#^	−8.67*^#^	< 0.001	−3.46	−3.50	−7.15*^#^	−6.27*^#^	0.003	−5.80	−9.12*	−9.16*	−10.60*	< 0.001
**△DBP (mmHg)**	−0.83	0.19*	− 1.86*^#^	− 1.12^#^	0.001	−0.35	1.02*	−2.49*^#^	−1.22^#^	< 0.001	− 1.43	− 0.74	− 1.51	− 1.03	0.515
**△BMI (kg/m**^**2**^**)**	0.63	− 0.48*	0.44^#^	−0.39*^$^	< 0.001	1.18	0.27*	1.52^#^	0.82*^#$^	< 0.001	−0.04	−1.34*	− 0.17^#^	− 1.36*^$^	< 0.001
**△WC (cm)**	3.89	3.05*	2.35*	2.01*^#^	< 0.001	4.15	2.93*	2.69*	1.55*^#^	< 0.001	3.58	3.21	2.15*^#^	2.38*	0.001
**△TC (mmol/L)**	−0.27	−0.22	−0.33^#^	−0.30^#^	0.069	−0.29	− 0.26	−0.32	− 0.34	0.469	− 0.25	−0.17	− 0.33^#^	−0.27^#^	0.068
**△TG (mmol/L)**	0.27	0.41*	0.04*^#^	0.12*^#^	< 0.001	0.26	0.40	−0.12*^#^	− 0.001*^#^	< 0.001	0.28	0.41	0.14^#^	0.22^#^	0.013
**△LDL-C (mmol/L)**	−0.10	− 0.14	− 0.06*^#^	− 0.10*^#$^	< 0.001	− 0.11	−0.13	− 0.10*	−0.11*^#$^	< 0.001	0.20	0.20	0.05*^#^	−0.001*^#^	< 0.001
**△HDL-C (mmol/L)**	−0.10	−0.14*	− 0.06^#^	−0.10^# $^	0.001	0.19	0.12	0.06	−0.11	0.668	−0.08	−0.15*	− 0.04*^#^	−0.10^#$^	< 0.001
**△FPG (mmol/L)**	0.02	0.09	−0.09	−0.05	0.072	0.07	0.11	−0.11	−0.04	0.262	−0.04	0.07	−0.08	− 0.06	0.354
**△eGFR (ml/min/1.73m**^**2**^**)**	−3.36	−5.12*	−1.48*^#^	−4.41*^$^	< 0.001	−4.27	−5.25	−1.76*^#^	−4.62	0.004	−2.25	− 4.98^#^	−1.31^#^	− 4.24^#$^	< 0.001

* *P* < 0.05, vs, MHNO; ^#^ P < 0.05, vs. MHO; ^$^ *P* < 0.005, vs. *MANO* MHNO metabolically health non-obese, *MHO* metabolically healthy obese, *MANO* metabolically abnormal obese, *MAO* metabolically abnormal obese

**Table 3:** Metabolic parameters of metabolic health non-obese (MHNO), metabolically healthy obese (MHO), metabolic abnormal non-obese (MANO) and metabolic abnormal obese (MAO) subjects during follow-up after adjusting for the baseline values

Total	MHNO	MHO	MANO	MAO	***P***-value
**SBP (mmHg)**	133.57 ± 0.34	134.35 ± 0.51	135.71 ± 0.76	136.52 ± 0.47*^#^	< 0.001
**DBP (mmHg)**	80.03 ± 0.19	82.03 ± 0.29^*^	81.14 ± 0.43	82.61 ± 0.27*^$^	< 0.001
**BMI (kg/m**^**2**^**)**	24.52 ± 0.08	25.24 ± 0.10^*^	24.71 ± 0.13^#^	25.78 ± 0.10*^#$^	< 0.001
**WC (cm)**	83.77 ± 0.16	86.31 ± 0.22^*^	84.10 ± 0.31^#^	87.14 ± 0.23*^#$^	< 0.001
**TC (mmol/L)**	4.79 ± 0.02	4.87 ± 0.02^*^	4.86 ± 0.04	4.92 ± 0.02*	< 0.001
**TG (mmol/L)**	1.59 ± 0.03	1.76 ± 0.05^*^	1.95 ± 0.07*	2.13 ± 0.04*^#^	< 0.001
**LDL-C (mmol/L)**	3.03 ± 0.01	3.07 ± 0.02	2.99 ± 0.03	2.95 ± 0.02*^#^	< 0.001
**HDL-C (mmol/L)**	1.42 ± 0.01	1.34 ± 0.02^*^	1.33 ± 0.02*	1.26 ± 0.01*^# $^	< 0.001
**FPG (mmol/L)**	5.74 ± 0.03	5.78 ± 0.05	5.95 ± 0.07*	6.00 ± 0.04*^#^	< 0.001
**eGFR (ml/min/1.73m**^**2**^**)**	98.98 ± 0.20	97.83 ± 0.30^*^	99.33 ± 0.45^#^	97.80 ± 0.27*^$^	< 0.001
**Men**	**MHNO**	**MHO**	**MANO**	**MAO**	***P*-value**
**SBP (mmHg)**	136.64 ± 0.45	138.56 ± 0.69	138.83 ± 1.26	139.97 ± 0.70^*^	0.001
**DBP (mmHg)**	82.14 ± 0.27	84.44 ± 0.40^*^	83.31 ± 0.74	84.67 ± 0.41^*^	< 0.001
**BMI (kg/m**^**2**^**)**	24.96 ± 0.10	25.88 ± 0.13^*^	25.67 ± 0.21^*^	27.00 ± 0.15^*#$^	< 0.001
**WC (cm)**	85.19 ± 0.22	87.76 ± 0.29^*^	85.94 ± 0.51^#^	88.91 ± 0.36^*#$^	< 0.001
**TC (mmol/L)**	4.80 ± 0.02	4.87 ± 0.03	4.87 ± 0.06	4.91 ± 0.03*	0.037
**TG (mmol/L)**	1.59 ± 0.05	1.82 ± 0.07^*^	2.21 ± 0.13^*#^	2.34 ± 0.07^*#^	< 0.001
**LDL-C (mmol/L)**	3.02 ± 0.02	3.03 ± 0.03	2.92 ± 0.05	2.87 ± .03^*#^	< 0.001
**HDL-C (mmol/L)**	1.42 ± 0.01	1.34 ± 0.01^*^	1.30 ± 0.03^*^	1.23 ± 0.01^*#^	< 0.001
**FPG (mmol/L)**	5.87 ± 0.04	5.88 ± 0.07	6.06 ± 0.12	6.19 ± 0.07^*#^	0.001
**eGFR (ml/min/1.73m**^**2**^**)**	97.43 ± 0.25	96.50 ± 0.39	99.08 ± 0.71^#^	96.77 ± 0.39^$^	0.007
**Women**	**MHNO**	**MHO**	**MANO**	**MAO**	***P*-value**
**SBP (mmHg)**	129.76 ± 0.50	129.67 ± 0.72	133.77 ± 0.93^*#^	133.87 ± 0.63^*#^	< 0.001
**DBP (mmHg)**	77.56 ± 0.27	79.41 ± 0.40^*^	79.52 ± 0.51^*^	80.89 ± 0.34^*#^	< 0.001
**BMI (kg/m**^**2**^**)**	23.95 ± 0.11	24.53 ± 0.14^*^	24.22 ± 0.16^#^	24.83 ± 0.13^*$^	< 0.001
**WC (cm)**	81.81 ± 0.25	85.01 ± 0.32^*^	82.64 ± 0.40^#^	85.99 ± 0.31^*$^	0.001
**TC (mmol/L)**	4.78 ± 0.023	4.89 ± 0.03	4.85 ± 0.043	4.93 ± 0.03^*^	0.001
**TG (mmol/L)**	1.58 ± 0.04	1.72 ± 0.05	1.74 ± 0.07^#^	1.90 ± 0.05^*^	< 0.001
**LDL-C (mmol/L)**	3.04 ± 0.02	3.11 ± 0.03	3.04 ± 0.04	3.02 ± 0.03	0.176
**HDL-C (mmol/L)**	1.42 ± 0.01	1.33 ± 0.01^*^	1.35 ± 0.02^*^	1.28 ± 0.01^*#$^	< 0.001
**FPG (mmol/L)**	5.60 ± 0.04	5.68 ± 0.08	5.84 ± 0.08	5.85 ± 0.05^*^	0.002
**eGFR (ml/min/1.73m**^**2**^**)**	100.83 ± 0.31	99.38 ± 0.46^*^	99.45 ± 0.59^#^	98.68 ± 0.38^*^	< 0.001

^*^ *P* < 0.05, vs, MHNO; ^#^ *P* < 0.05, vs. MHO; ^$^ *P* < 0.005, vs. *MANO* MHNO metabolically health non-obese, *MHO* metabolically healthy obese, *MANO* metabolically abnormal obese, *MAO* metabolically abnormal obese

### Association between mildly reduced eGFR and obese phenotype in different gender

In Table 4, we show the association between mildly reduced eGFR and MHO. After adjusting for possible confounders, MHO was associated with a higher cumulative incidence of mildly reduced eGFR in both men [OR (95% CI): 1.62 (1.32, 1.98)] and women [OR (95% CI): 1.63 (1.18, 2.25)]. Furthermore, MAO in men also increased the risk of mildly reduced eGFR compared to MHNO [OR (95% CI): 1.74 (1.32, 2.29)].

**Table 4:** Association between mildly reduced eGFR and obese phenotype in different gender

	Model 1		Model 2		Model 3	***P***-value
	OR (95%CI)	***P***-value	OR (95%CI)	***P***-value	OR (95%CI)	
**Total**						
**MHNO**	1.00(reference)		1.00(reference)		1.00(reference)	
**MHO**	1.35 (1.11,1.64)	0.002	1.63 (1.33,1.99)	< 0.001	1.62 (1.32,1.98)	< 0.001
**MANO**	1.11 (0.84,1.46)	0.453	0.89 (0.67,1.19)	0.438	0.87 (0.65,1.17)	0.280
**MAO**	1.43 (1.19,1.71)	< 0.001	1.48 (1.22,1.78)	< 0.001	1.44 (1.17,1.75)	< 0.001
**Women**						
**MHNO**	1.00(reference)		1.00(reference)		1.00(reference)	
**MHO**	1.36 (1.01,1.83)	0.045	1.65 (1.20,2.27)	0.002	1.63 (1.18,2.25)	0.003
**MANO**	1.48 (1.05,2.09)	0.026	0.98 (0.67,1.41)	0.920	0.97 (0.66,1.41)	0.860
**MAO**	1.43 (1.10,1.87)	0.008	1.17 (0.88,1.55)	0.309	1.18 (0.87,1.59)	0.290
**Men**						
**MHNO**	1.00 (reference)		1.00 (reference)		1.00 (reference)	
**MHO**	1.35 (1.05,1.74)	0.021	1.59 (1.22,2.07)	0.001	1.62 (1.24,2.11)	0.001
**MANO**	0.67 (0.41,1.09)	0.106	0.61 (0.37,1.01)	0.053	0.61 (0.37,1.01)	0.055
**MAO**	1.45 (1.13,1.86)	0.003	1.72 (1.33,2.23)	< 0.001	1.74 (1.32,2.29)	< 0.001

Model 1. Unadjusted; Model 2. Adjusted for age, current smoking, current drinking (in addition to gender in total group); Model 3. Adjusted for age, current smoking, current drinking, chronic diseases, *LDL-C* ALT, AST (in addition to gender in total group), *MHNO* metabolically health non-obese, *MHO* metabolically healthy obese, *MANO* metabolically abnormal obese, *MAO* metabolically abnormal obese

## Discussion

In the present study, the prevalence of MHNO, MHO, MANO and MAO among rural Northeast China residents was 46.5, 20.0, 9.1 and 24.4%, respectively. Meanwhile, the cumulative incidence of mildly reduced eGFR among participants with MHNO, MHO, MANO and MAO was 15.5, 20.1, 16.7 and 20.8%, respectively. A high proportion of subjects experienced obese metabolic phenotype changes during the follow-up time. After adjusting for possible confounders, MHO was associated with a higher cumulative incidence of mildly reduced eGFR among women and men. Furthermore, MAO was associated with a mild decrease in eGFR among men only.

Renal dysfunction is closely related to many cardiovascular diseases (CVDs) and is associated with higher morbidity and mortality [5]. At first, many studies focused on severe chronic kidney diseases characterized by extremely low eGFR. However, as growing concern was put on mild reductions in eGFR, cumulative evidence confirmed that mild renal dysfunction also correlated with a higher risk of CVD and cerebrovascular diseases [5]. Recently, a study reported that eGFR was significantly correlated with slow coronary flow in patients with normal to mildly impaired renal function [26]. Furthermore, Khurram Nasir and colleagues reported that impaired regional systolic and diastolic function was observed among subjects with mild and moderated reductions of renal function without clinical heart diseases [27]. Hence, it is necessary to routinely evaluate renal function to identify subjects with early cardiovascular risk. The possible explanations for why mildly reduced eGFR increases CVD risk remain controversial, but some possible reasons are proposed. Masanobu Yoshida concluded that mildly reduced eGFR was associated with increased arterial stiffness, which acts as a definite risk factor for CVD [28]. In addition, another study reported that endothelial dysfunction contributed to the excess cardiovascular mortality in subjects with mild renal insufficiency [29]. Similarly, oxidative stress, the imbalance between prooxidant/antioxidant processes, resulted in an increase in reactive oxygen species, which diminished the expression of antioxidant enzymes and caused renal dysfunction [30]. In our study, the cumulative incidence of mildly reduced eGFR was 17.97%, which was higher than estimates from other previous studies also held in Asia [31]. Therefore, early detection and screening of the possible risk factors for mildly decreased kidney function is an important strategy to reduce chronic kidney diseases.

In the present study, the prevalence of MHO at baseline was 19.0% among women and 21.1% among men. Among 11,465 men and 16,612 women in Europe, the age-standardized prevalence of MHO was 12% across all cohorts [32]. The highest prevalence of MHO among men was 19% in the CHRIS study [32]. There was a sex difference in the prevalence of MHO in other studies. In the NCDS from the UK, men had a significantly lower rate of MHO than women (9% vs. 28.4%) [33]. Similar differences have been found by earlier studies in Caucasian, Asian and African American subjects [34]. However, there was a lack of sex differences in the prevalence of MHO in our present study, and the relatively higher prevalence of MHO at baseline might be due to differences in the definition. The prevalence of MHO in the present study was based on the WHO Asia Pacific obesity guidelines definition and used BMI ≥ 25 kg/m^2^ as the threshold, as has been done in many previous studies; however this still underestimates the prevalence of MHO among rural Northeast residents [35, 36]. Several mechanisms might be relevant to this obese phenotype, such as maintenance of insulin sensitivity, the specific fat distribution, normal adipose tissue function and a normal adipokine secretion pattern [33, 37]. In recent years, it was debated whether individuals with MHO are truly healthy, especially if there is a lack of general agreement on unified criteria to define MHO. Furthermore, subjects with MHO did not obtain significant improvement in their cardiovascular risk factors upon weight loss interventions and therefore might not benefit to the same extent as subjects with MAO [38]. It is even harder for subjects with MHO to control their risk of developing CVD. For the MHO-related risk factors, cumulative evidence confirmed the association between MHO and renal dysfunction. Some reported that subjects with persistent MHO had a 2-fold increased risk of chronic kidney disease [39], whereas others claimed that metabolic abnormalities, but not obesity, caused a mild decrease in eGFR [40]. In our study, we found that MHO was associated with a higher cumulative incidence of mildly reduced eGFR in both women and men. This underscores the possible effect of MHO on renal function. Interestingly, MAO was associated with a higher incidence of mildly reduced eGFR among men but not women. There was a study intending to determine the mechanism of the different metabolic characteristics of obesity that concluded that the metabolite panel, including L-kynurenine, glycerophosphocholine (GPC), glycerol 1-phosphate, glycolic acid and uric acid levels, was significantly different between MHO and MAO groups [41]. There might be some metabolic differences between women and men that make MAO associated with mild kidney dysfunction among men but not women.

### Limitation

First, due to the lack of uniform criteria for defining metabolic healthy obesity, the rate of metabolic healthy obesity might have varied results, which makes the conclusion biased. However, in the present study, we chose the relatively widely used definition [25]. Second, the calculation of eGFR was based on a single blood test assessment, which might introduce bias. Third, even though we excluded those with renal diseases at baseline, we did not adjust for some factors that might affect eGFR, such as medication use. Fourth, using the CKD-EPI equation to calculate eGFR to estimate GFR might not be accurate.

## Conclusion

In the present study, we reported a relatively high prevalence of MHO and other obese phenotypes at baseline. In addition, the changes in the obese metabolic phenotypes over time were dramatic, and more emphasis should be placed on the abnormal phenotypes. MHO was associated with a higher cumulative incidence of mildly reduced eGFR among women and men, while MAO correlated with a mild decrease in eGFR only among men. Routine screening of kidney function should be recommended among subjects with MHO among rural Northeast China.

## Data Availability

Enquiries regarding the availability of primary data should be directed to the principal investigator Professor Yingxian Sun (sunyingxiancmu1h@163.com).

## References

[1] Astor BC, Hallan SI, Miller ER, Yeung E, Coresh J (2008). Glomerular filtration rate, albuminuria, and risk of cardiovascular and all-cause mortality in the US population. Am J Epidemiol.

[2] Manjunath G, Tighiouart H, Ibrahim H, MacLeod B, Salem DN, Griffith JL, Coresh J, Levey AS, Sarnak MJ (2003). Level of kidney function as a risk factor for atherosclerotic cardiovascular outcomes in the community. J Am Coll Cardiol.

[3] Brugts JJ, Knetsch AM, Mattace-Raso FU, Hofman A, Witteman JC (2005). Renal function and risk of myocardial infarction in an elderly population: the Rotterdam study. Arch Intern Med.

[4] Kurth T, de Jong PE, Cook NR, Buring JE, Ridker PM (2009). Kidney function and risk of cardiovascular disease and mortality in women: a prospective cohort study. BMJ (Clin Res ed).

[5] Polonsky TS, Locatelli F (2010). The contribution of early nephropathy to cardiovascular risk. Cardiol Clin.

[6] Ford ND, Patel SA, Narayan KM (2017). Obesity in low- and middle-income countries: burden, drivers, and emerging challenges. Annu Rev Public Health.

[7] Popkin BM, Adair LS, Ng SW (2012). Global nutrition transition and the pandemic of obesity in developing countries. Nutr Rev.

[8] Sainju NK, Shah RK, Joshi SK (2018). Screening for hypertension and obesity in rural population of Nepal. Kathmandu Univ Med J (KUMJ).

[9] Rai RK, Jaacks LM, Bromage S, Barik A, Fawzi WW, Chowdhury A (2018). Prospective cohort study of overweight and obesity among rural Indian adults: sociodemographic predictors of prevalence, incidence and remission. BMJ Open.

[10] Trivedi T, Liu J, Probst J, Merchant A, Jhones S, Martin AB (2015). Obesity and obesity-related behaviors among rural and urban adults in the USA. Rural Remote Health.

[11] He Y, Pan A, Wang Y, Yang Y, Xu J, Zhang Y, Liu D, Wang Q, Shen H, Zhang Y (2017). Prevalence of overweight and obesity in 15.8 million men aged 15–49 years in rural China from 2010 to 2014. Sci Rep.

[12] Tian H, Xie H, Song G, Zhang H, Hu G (2009). Prevalence of overweight and obesity among 2.6 million rural Chinese adults. Prev Med.

[13] Silva Junior GB, Bentes AC, Daher EF, Matos SM (2017). Obesity and kidney disease. J Bras Nefrol.

[14] Aguilar-Salinas CA, García EG, Robles L, Riaño D, Ruiz-Gomez DG, García-Ulloa AC, Melgarejo MA, Zamora M, Guillen-Pineda LE, Mehta R (2008). High adiponectin concentrations are associated with the metabolically healthy obese phenotype. J Clin Endocrinol Metab.

[15] Wildman RP, Muntner P, Reynolds K, McGinn AP, Rajpathak S, Wylie-Rosett J, Sowers MR (2008). The obese without cardiometabolic risk factor clustering and the normal weight with cardiometabolic risk factor clustering: prevalence and correlates of 2 phenotypes among the US population (NHANES 1999-2004). Arch Intern Med.

[16] Hamer M, Stamatakis E (2012). Metabolically healthy obesity and risk of all-cause and cardiovascular disease mortality. J Clin Endocrinol Metab.

[17] Appleton SL, Seaborn CJ, Visvanathan R, Hill CL, Gill TK, Taylor AW, Adams RJ (2013). Diabetes and cardiovascular disease outcomes in the metabolically healthy obese phenotype: a cohort study. Diabetes Care.

[18] Yu S, Guo X, Yang H, Zheng L, Sun Y (2014). An update on the prevalence of metabolic syndrome and its associated factors in rural Northeast China. BMC Public Health.

[19] Li Z, Guo X, Zheng L, Yang H, Sun Y (2015). Grim status of hypertension in rural China: results from Northeast China rural cardiovascular health study 2013. J Am Soc Hypertension.

[20] Panagiotakos DB, Pitsavos C, Chrysohoou C, Risvas G, Kontogianni MD, Zampelas A, Stefanadis C (2004). Epidemiology of overweight and obesity in a Greek adult population: the ATTICA study. Obes Res.

[21] Chobanian AV, Bakris GL, Black HR, Cushman WC, Green LA, Izzo JL, Jones DW, Materson BJ, Oparil S, Wright JT (2003). The seventh report of the joint National Committee on prevention, detection, evaluation, and treatment of high blood pressure: the JNC 7 report. JAMA.

[22] Levey AS, Stevens LA, Schmid CH, Zhang YL, Castro AF, Feldman HI, Kusek JW, Eggers P, Van Lente F, Greene T (2009). A new equation to estimate glomerular filtration rate. Ann Intern Med.

[23] Sung KC, Cha SC, Sung JW, So MS, Byrne CD (2014). Metabolically healthy obese subjects are at risk of fatty liver but not of pre-clinical atherosclerosis. Nutr Metab Cardiovasc Dis.

[24] Alberti KG, Eckel RH, Grundy SM, Zimmet PZ, Cleeman JI, Donato KA, Fruchart JC, James WP, Loria CM, Smith SC (2009). Harmonizing the metabolic syndrome: a joint interim statement of the international diabetes federation task force on epidemiology and prevention; National Heart, Lung, and Blood Institute; American Heart Association; world heart federation; international atherosclerosis society; and International Association for the Study of obesity. Circulation.

[25] Latifi SM, Karandish M, Shahbazian H, Taha JM, Cheraghian B, Moradi M (2017). Prevalence of metabolically healthy obesity (MHO) and its relation with incidence of metabolic syndrome, hypertension and type 2 diabetes amongst individuals aged over 20 years in Ahvaz: a 5 year cohort study (2009-2014). Diab Metab Syndrome.

[26] Akin F, Celik O, Altun I, Ayça B (2014). Association of glomerular filtration rate with slow coronary flow in patients with normal to mildly impaired renal function. Angiology.

[27] Nasir K, Rosen BD, Kramer HJ, Edvardsen T, Bluemke DA, Liu K, Lima JA (2007). Regional left ventricular function in individuals with mild to moderate renal insufficiency: the multi-ethnic study of atherosclerosis. Am Heart J.

[28] Yoshida M, Tomiyama H, Yamada J, Koji Y, Shiina K, Nagata M, Yamashina A (2007). Relationships among renal function loss within the normal to mildly impaired range, arterial stiffness, inflammation, and oxidative stress. Clin J Am Soc Nephrol.

[29] Stam F, van Guldener C, Becker A, Dekker JM, Heine RJ, Bouter LM, Stehouwer CD (2006). Endothelial dysfunction contributes to renal function-associated cardiovascular mortality in a population with mild renal insufficiency: the Hoorn study. J Am Soc Nephrol.

[30] Miranda-Díaz AG, Pazarín-Villaseñor L, Yanowsky-Escatell FG, Andrade-Sierra J: Oxidative stress in diabetic nephropathy with early chronic kidney disease. J Diab Res 2016, 2016:7047238. 10.1155/2016/7047238 PMC4971321 27525285

[31] Toyama T, Furuichi K, Shimizu M, Hara A, Iwata Y, Sakai N, Perkovic V, Kobayashi M, Mano T, Kaneko S (2015). Relationship between serum uric acid levels and chronic kidney disease in a Japanese cohort with Normal or mildly reduced kidney function. PLoS One.

[32] van Vliet-Ostaptchouk JV, Nuotio ML, Slagter SN, Doiron D, Fischer K, Foco L, Gaye A, Gögele M, Heier M, Hiekkalinna T (2014). The prevalence of metabolic syndrome and metabolically healthy obesity in Europe: a collaborative analysis of ten large cohort studies. BMC Endocr Disord.

[33] Blüher M (2012). Are there still healthy obese patients?. Curr Opinion Endocrinol Diab Obes.

[34] Pajunen P, Kotronen A, Korpi-Hyövälti E, Keinänen-Kiukaanniemi S, Oksa H, Niskanen L, Saaristo T, Saltevo JT, Sundvall J, Vanhala M (2011). Metabolically healthy and unhealthy obesity phenotypes in the general population: the FIN-D2D survey. BMC Public Health.

[35] Hwang LC, Bai CH, Sun CA, Chen CJ (2012). Prevalence of metabolically healthy obesity and its impacts on incidences of hypertension, diabetes and the metabolic syndrome in Taiwan. Asia Pac J Clin Nutr.

[36] Cherqaoui R, Kassim TA, Kwagyan J, Freeman C, Nunlee-Bland G, Ketete M, Xu S, Randall OS (2012). The metabolically healthy but obese phenotype in African Americans. J Clin Hypertension (Greenwich, Conn).

[37] Stefan N, Häring HU, Hu FB, Schulze MB (2013). Metabolically healthy obesity: epidemiology, mechanisms, and clinical implications. Lancet Diab Endocrinol.

[38] Blüher M (2014). Are metabolically healthy obese individuals really healthy?. Eur J Endocrinol.

[39] Nam KH, Yun HR, Joo YS, Kim J, Lee S, Lee C, Park KS, Park JT, Chang TI, Kang EW (2018). Changes in obese metabolic phenotypes over time and risk of incident chronic kidney disease. Diabetes Obes Metab.

[40] Wang C, Liang K, Zhang X, Li C, Yang W, Ma Z, Sun Y, Song J, Lin P, Gong L (2014). Metabolic abnormalities, but not obesity, contribute to the mildly reduced eGFR in middle-aged and elderly Chinese. Int Urol Nephrol.

[41] Chen HH, Tseng YJ, Wang SY, Tsai YS, Chang CS, Kuo TC, Yao WJ, Shieh CC, Wu CH, Kuo PH (2015). The metabolome profiling and pathway analysis in metabolic healthy and abnormal obesity. Int J Obes (2005).

